# miR-27a regulates cisplatin resistance and metastasis by targeting RKIP in human lung adenocarcinoma cells

**DOI:** 10.1186/1476-4598-13-193

**Published:** 2014-08-16

**Authors:** Jipeng Li, Yiping Wang, Yulan Song, Zhongming Fu, Wanjun Yu

**Affiliations:** Department of Clinical Laboratory, Yinzhou People’s Hospital, Ningbo, 315040 China; Department of Nephrology, Yinzhou People’s Hospital, Ningbo, 315040 China; Department of Respiratory and Critical Care Medicine, Yinzhou People’s Hospital, 251 East Baizhang Road, Ningbo, 315040 China

**Keywords:** miR-27a, RKIP, Lung adenocarcinoma, Cisplatin, Chemoresistance

## Abstract

**Background:**

MicroRNAs (miRNAs) have been identified as important posttranscriptional regulators involved in various biological and pathological processes of cells, but their association with tumor chemoresistance has not been fully understood.

**Methods:**

We detected miR-27a expression in two lung adenocarcinoma cell lines, A549 and A549/CDDP, and then investigated the effects of miR-27a on the metastasis and the chemosensitivity of cancer cells, using both gain- and loss-of-function studies. The correlation between miR-27a level and chemoresistance was further investigated in clinical lung adenocarcinoma specimens.

**Results:**

miR-27a was significantly up-regulated in cisplatin-resistant lung adenocarcinoma A549/CDDP cells compared with parental A549 cells. miR-27a regulates epithelial-mesenchymal transition (EMT) and cisplatin resistance *in vitro* and modulates response of lung adenocarcinoma cells to cisplatin *in vivo*. Further studies identified Raf Kinase Inhibitory Protein (RKIP) as a direct and functional target of miR-27a. Small interfering RNA-mediated RKIP knockdown revealed similar effects as that of ectopic miR-27a expression, while overexpression of RKIP attenuated the function of miR-27a in lung adenocarcinoma cells. Increased miR-27a expression was also detected in tumor tissues sampled from lung adenocarcinoma patients treated with cisplatin-based chemotherapy and was proved to be correlated with low expression of RKIP, decreased sensitivity to cisplatin, and poor prognosis.

**Conclusion:**

Our results suggest that up-regulation of miR-27a could suppress RKIP expression and in turn contribute to chemoresistance of lung adenocarcinoma cells to cisplatin.

**Electronic supplementary material:**

The online version of this article (doi:10.1186/1476-4598-13-193) contains supplementary material, which is available to authorized users.

## Background

Lung cancer is the leading cause of cancer-related death among men and women worldwide, Approximately 70%-80% of lung cancers are non-small cell lung cancer (NSCLC), including squamous cell carcinoma, adenocarcinoma, and large cell carcinoma [1, 2]. In NSCLC, the leading death cause is chemotherapy resistance and metastasis, yet the underlying mechanisms of them remain largely unclear [3–6]. Previous studies in ovarian carcinoma, breast cancer, colorectal cancer and tongue cancer have demonstrated that the drug-resistant cancer cells display features of epithelial-mesenchymal transition (EMT), characterized by the loss of the epithelial marker E-cadherin, an increase in the mesenchymal markers vimentin and N-cadherin, and an increase in the invasion and metastasis behavior [7–11]. Therefore, chemotherapy-induced EMT in tumor cells has been linked to chemotherapeutic resistance and metastasis.

MicroRNAs (miRNAs) are a class of small noncoding RNA molecules that negatively regulate the expression of target genes by either mRNA degradation or translational inhibition. miRNAs are involved in various biological and pathological processes such as differentiation, morphogenesis, and carcinogenesis [12–14]. Accumulating evidence has demonstrated that miRNAs have a key role in drug resistance and EMT. For example, miR-141 is reported to enhance cisplatin resistance through repression of KEAP1 in ovarian cancer cells [15], miR-106a induces multidrug resistance in gastric cancer by targeting RUNX3 [16]. In contrast, MiR-200b and miR-15b reverse chemotherapy-induced EMT in human tongue cancer cells by targeting BMI1 [10]. The involvement of miRNAs in drug resistance is just beginning to emerge, and more studies are needed to identify other miRNAs, their molecular targets and the processes they affect.

In this study, we observed that miR-27a is significantly upregulated in cisplatin-resistant human lung adenocarcinoma A549/CDDP cells compared with parental A549 cells. Next we explored the roles of miR-27a and its target Raf Kinase Inhibitory Protein (RKIP) in regulating cisplatin resistance and metastasis in lung adenocarcinoma. Finally, we correlated the expression of miR-27a and RKIP with the chemotherapeutic status and prognosis of lung adenocarcinoma patients. Our results show that miR-27a has the potential as key regulatory factors for the chemotherapy resistance and metastasis of lung adenocarcinoma.

## Results

### Parental A549 cells and cisplatin-resistant A549/CDDP cells differ in morphology, physiology, and miRNA expression

**Figure 1:**
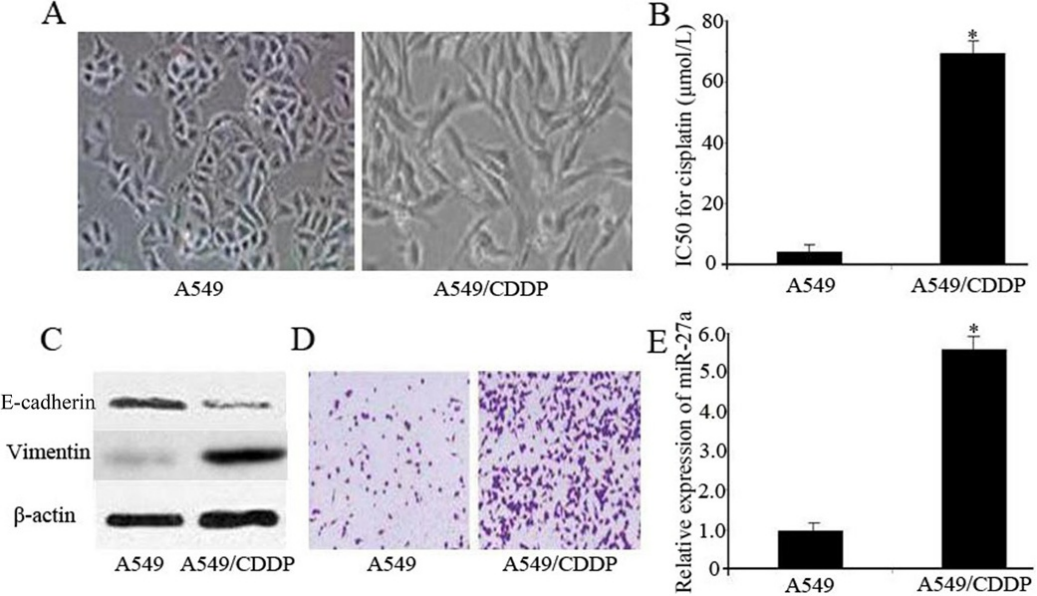
**Differences between A549/CDDP cells and parental A549 cells are shown. (A)** A549/CDDP cells exhibiting fibroblastic morphology and the A549 cells showing epithelial-like appearance (original magnification, ×100). **(B)** MTT assay shows that A549/CDDP cells are much more resistant to cisplatin than A549 cells. **(C)** Western blotting illustrates reduced expression of E-cadherin and increased expression of vimentin in A549/CDDP cells. β-actin was used as an internal control. **(D)** Invasion assay reveals significant enhancement of invasion ability of A549/CDDP cells in *vitro*. **(E)** qRT-PCR indicates a significant up-regulation of miR-27a in A549/CDDP cells compared with A549 cells. miRNA abundance was normalized to U6 RNA. Data are means of three separated experiments ± SD; *P < 0.01.

To better understand the biological mechanisms of chemoresistance in lung adenocarcinoma cells and search for the reversion opportunities, we made use of a cisplatin-sensitive and derived resistant lung cancer cell line pair (A549 and A549/ CDDP). Compared with parental A549 cells, A549/CDDP cells displayed spindle shape and separated from one other (Figure 1A), MTT assay was then performed, and the IC50 of A549/CDDP cells increased 15.7 fold (Figure 1B). Moreover, western blotting demonstrated that the protein expression of E-cadhein decreased, while that of vimentin dramatically increased in A549/CDDP cells (Figure 1C). Additionally, invasion assay demonstrated that the invasion significantly increased in A549/CDDP as compared with A549 cells (Figure 1D).

Based on the miRNA microarray data, 13 miRNAs were found to be differentially expressed (>2-fold change) in A549/CDDP cells compared with A549 cells (Additional file 1: Table S1), among which miR-27a was the most up-regulated one (5.6-fold change). The result was validated via real-time quantitative RT-PCR (Figure 1E).

### miR-27a promotes EMT and cisplatin resistance *in vitro*

**Figure 2:**
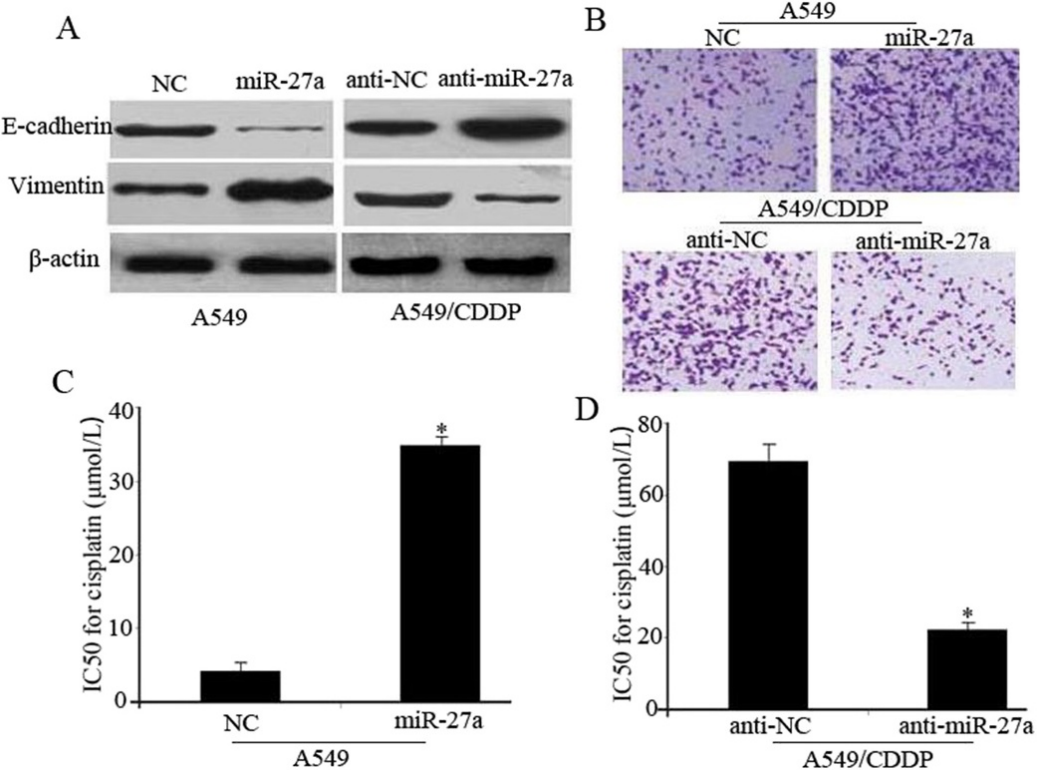
**miR-27a promotes EMT and cisplatin resistance** ***in vitro*** **.** A549 cells were transfected with NC or miR-27a mimics, A549/CDDP cells were transfected with anti-NC or miR-27a inhibitors respectively. Western blotting was used to detect E-cadherin and vimentin expression, β-actin was used as an internal control **(A)**. transwell invasion assay **(B)** and MTT assay **(C** and **D)** were used to measure invasion ability and cisplatin sensitivity. Data are means of three separated experiments ± SD; *P < 0.01.

To investigate the association of miR-27a expression with lung adenocarcinoma chemoresistance against cisplatin, A549 and A549/CDDP cells were transfected with miR-27a mimics and miR-27a inhibitors respectively. As shown in Figure 2A, miR-27a mimics increased vimentin, but suppressed E-cadherin expression in A549 cells. Invasion assay demonstrated that miR-27a mimics synergistically enhanced the invasion of A549 cells (Figure 2B). On the other hand, silencing miR-27a expression in A549/CDDP cells using anti-sense oligonucleotides reduced vimentin and increased the expression of E-cadherin (Figure 2A). Furthermore, miR-27a inhibitors synergistically suppressed the invasion of A549/CDDP cells (Figure 2B). Therefore, upregulation of miR-27a may play an important role in chemotherapy-induced EMT of lung adenocarcinoma cells. Moreover, MTT assay revealed that A549 cells transfected with miR-27a mimics showed greatly decreased sensitivity to cisplatin as indicated by substantially increased IC50 values (Figure 2C). In contrast, suppression of the miR-27a level in A549/CDDP cells resulted in an enhanced sensitivity to cisplatin (Figure 2D). Similar results were obtained in H1395 and H1299 cells (Additional file 2: Figure S1).

### miR-27a regulates response of lung adenocarcinoma cells to cisplatin *in vivo*

**Figure 3:**
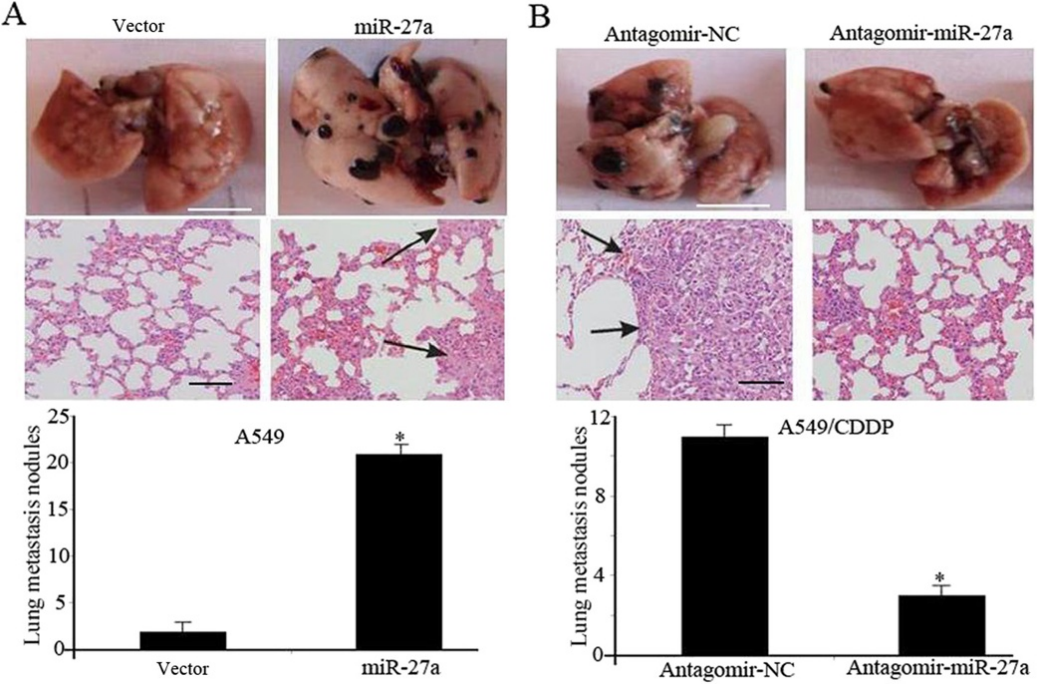
**miR-27a regulates response of lung adenocarcinoma cells to cisplatin** ***in vivo.*** **(A)** A549 cells were performed after transduction by miR-27a-expressing or vector lentivirus. Representative images of nude mouse lungs (scale bars: 5 mm) and H&E stain of lungs (scale bars: 100 μm) are shown. **(B)** A549/CDDP cells were performed after transduction by miR-27a-inhibiting or antagomir-NC. Representative images of nude mouse lungs (scale bars: 5 mm) and H&E stain of lungs (scale bars: 100 μm) are shown. *P < 0.01.

To investigate the effect of miR-27a expression on chemosensitivity of lung adenocarcinoma *in vivo*, A549 cells stably expressing miR-27a by lentivirus were subcutaneously inoculated into nude mice. When the average tumor size reached ≈ 50 mm^3^, cisplatin was administered via intraperitoneal injection at a dose of 5 mg/kg, 1 dose every other day, with 3 doses in total. As shown in Figure 3A, the number of lung metastasis nodules was dramatically increased in miR-27a overexpression group when compared with control. Next, we engineered A549/CDDP cells to stably inhibit miR-27a with a lentivirus-mediated antagomir. The results showed that suppression of miR-27a decreased the number of lung metastases (Figure 3B). These results suggested that miR-27a could regulate the response to cisplatin *in vivo.*

### miR-27a directly targets RKIP

**Figure 4:**
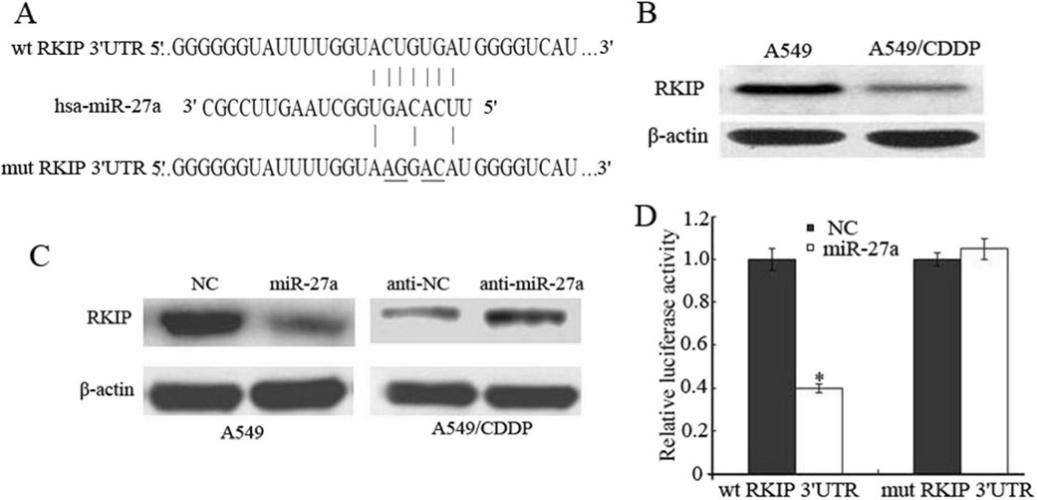
**RKIP is a direct target of miR-27a. (A)** The predicted miR-27a binding site within RKIP 3′UTR and its mutated version by site mutagenesis are as shown. **(B)** Variable RKIP expression in A549 and A549/CDDP was obtained by western blot. **(C)** A549 cells were transfected with NC or miR-27a mimics, A549/CDDP cells were transfected with anti-NC or miR-27a inhibitors respectively. Western blotting was used to detect RKIP expression; β-actin was used as an internal control. **(D)** Luciferase assay was performed in A549 cells that were co-transfected with miRNA mimics and reporter vectors carrying RKIP 3′ UTR with wild type versus mutated miR-27a response element. Data are means of three separated experiments ± SD; *P < 0.01.

By employing open access softwares (TargetScan and PicTarget), RKIP was chosen as a preferred candidate target gene of miR-27a because of the putative binding site within its 3′UTR (Figure 4A) and lower RKIP protein expression in A549/CDDP cells (Figure 4B). Western blot showed that overexpression of miR-27a in A549 cells significantly repressed RKIP protein expression compared to cells transfected with negative control (Figure 4C). Relatively, downregulation of miR-27a by inhibitors in A549/CDDP cells led to a moderate increase of RKIP protein level (Figure 4C). To verity whether RKIP is the direct downstream target of miR-27a, a fragment of RKIP 3′UTR containing the putative miR-27a binding site was cloned into a luciferase reporter vector. Luciferase reporter assays showed that up-regulation of miR-27a significantly decreased the relative luciferase activity of RKIP 3′UTR in A549 cells, but had no effect on the mutant of RKIP 3′UTR (Figure 4D). Taken together, these results suggest that miR-27a down-regulates RKIP expression by directly targeting its 3′UTR.

### RKIP is involved in miR-27a-induced EMT and cisplatin resistance

**Figure 5:**
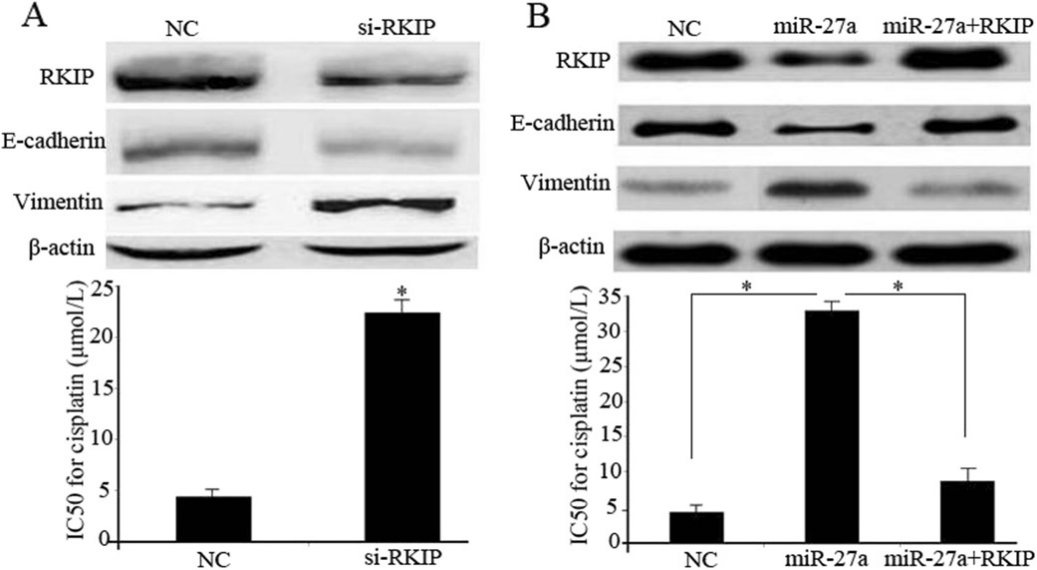
**RKIP is involved in miR-27a-induced EMT and cisplatin resistance. (A)** A549 cells were transfected with RKIP siRNAs, then RKIP, E-cadherin and vimentin protein levels were detected by western blot analysis. β-actin was used as an internal control. MTT assays were used to measure cisplatin sensitivity. **(B)** A549 cells were transfected with NC, miR-27a mimics or plasmid lacking 3′UTR along with miR-27a, RKIP, E-cadherin and vimentin protein levels were detected by western blot analysis. β-actin was used as an internal control. MTT assays were used to measure cisplatin sensitivity. Data are means of three separated experiments ± SD; *P < 0.01.

To further examine whether RKIP is involved in miR-27a-induced chemoresistance, we performed loss-of-function and gain-of-function analyses. Firstly, A549 cells were transfected with si-RKIP or negative control. Western blotting analysis confirmed that the expression of RKIP was suppressed (Figure 5A). As expected, RKIP knockdown significantly increased vimentin, reduced E-cadherin and decreased sensitivity to cisplatin in A549 cells (Figure 5A). Subsequently, we employed an expression construct that encodes the entire RKIP coding sequence but lacks the 3′UTR. Ectopic expression of RKIP partially rescued miR-27a-mediated EMT and cisplatin resistance in miR-27a-overexpressing cells (Figure 5B). Collectively, these data suggest that miR-27a regulate chemoresistance of lung adenocarcinoma cells at least in part by targeting RKIP.

### High expression of miR-27a in lung adenocarcinoma tissues is associated with decreased RKIP expression, chemotherapeutic resistance, and poor prognosis

**Figure 6:**
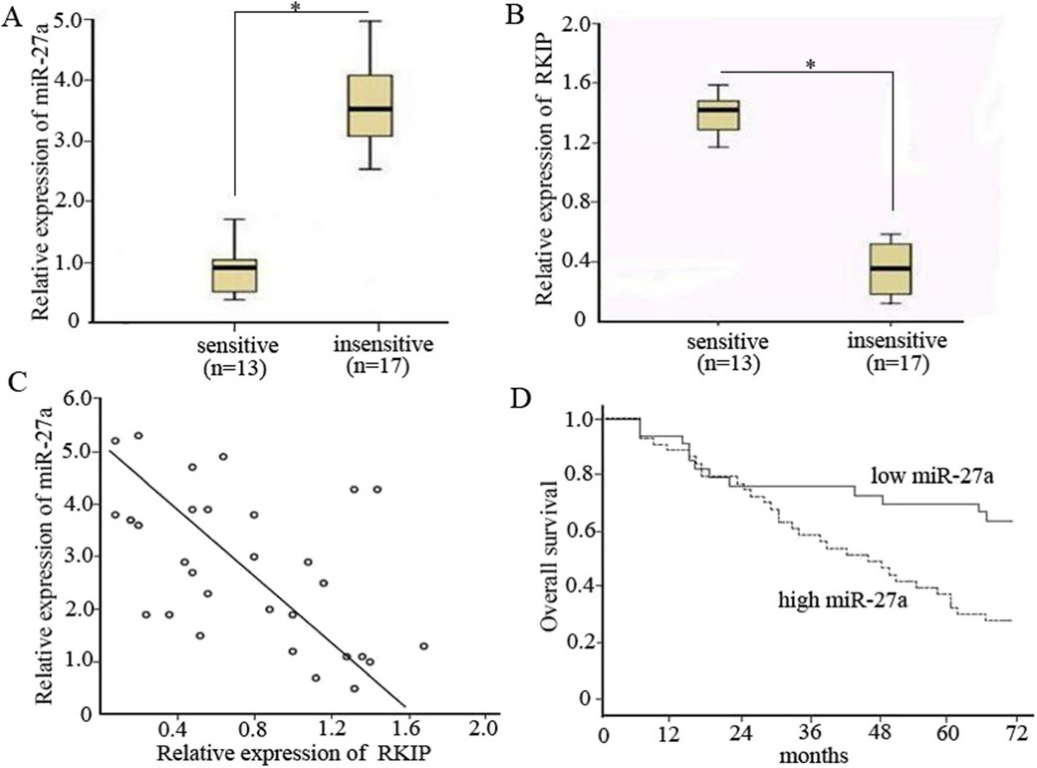
**The inverse correlation between miR-27a and RKIP expression in lung adenocarcinoma tissue samples and the clinical significance of miR-27a are shown.** Relative expression levels of **(A)** miR-27a and **(B)** RKIP mRNA were detected in cisplatin-sensitive (n =13) and insensitive (n = 17) lung adenocarcinoma tissues via qRT-PCR. Abundance of miRNA and RKIP mRNA was normalized to U6 RNA and GAPDH, respectively. **(C)** Expression levels of miR-27a and RKIP mRNA are inversely correlated among all the tissue samples (n = 30) as indicated by two-tailed Pearson’s correlation analysis, r = −0.691; p < 0.01. **(D)** Kaplan-Meier survival curve indicates that patients with high miR-27a expression have shorter overall survival than those with low miR-27a expression (log-rank test, *P <* 0.01).

To better understand the association between miR-27a and RKIP expression, a total of 30 clinical tumor tissue samples were collected from patients with advanced lung adenocarcinoma and divided into^“^sensitive^”^ and ^“^insensitive^”^groups according to the patient’s response to cisplatin-based chemotherapy. As shown in Figure 6A, miR-27a was significantly up-regulated in the^“^insensitive^”^group tissues (n = 17) compared with that in the ^“^sensitive^”^group ones (n = 13). On the contrary, RKIP mRNA expression level was significantly down-regulated in the^“^insensitive^”^ group tissues (Figure 6B). The inverse correlation between miR-27a and RKIP mRNA expression was verified by linear regression analysis (r = −0.691, P < 0.01) (Figure 6C). We then analyzed the association of miR-27a expression with survival of patients. As shown in Figure 6D, Patients with high miR-27a expression showed significantly shorter overall survival than those with low miR-27a expression (P < 0.01).

## Discussion

In the current study, we demonstrate that upregulation of miR-27a is critical for cisplatin resistance and tumor metastasis of lung adenocarcinoma cells both *in vitro* and *in vivo*, and miR-27a induces mesenchymal features and promotes tumor metastasis of chemoresistant lung adenocarcinoma via silencing RKIP. Furthermore, upregulation of miR-27a is correlated with cisplatin resistance and poor prognosis of lung adenocarcinoma patients. These findings provide new insights into the molecular functions of miR-27a as well as the role of RKIP in chemotherapeutic resistance.

MiR-27a is located at chromosome 19 and has been shown to be overexpressed in breast cancer, gastric cancer and cervical cancer [17–19]. In gastric cancer cells, miR-27a promots cell growth and metastasis both *in vitro* and *in vivo* [20, 21]. In addition, it could regulate endothelial differentiation of breast cancer stem like cells [22]. Moreover, miR-27a plays an important role in mediating drug resistance by targeting multiple drug-resistance related genes. MiR-27a modulated MDR1/P-glycoprotein expression in human ovarian cancer cells by targeting HIPK2 [23] and Down-regulation of miR-27a might reverse multidrug resistance of esophageal squamous cell carcinoma through regulation of MDR1 and apoptosis [24]. Despite the oncogenic role of miR-27a has been implicated by previous studies, the role of miR-27a in lung cancer chemotherpy and molecular mechanisms are not known. Here we identified RKIP as the functional target, through which miR-27a regulates metastasis and chemoresistance.

Raf Kinase Inhibitory Protein (RKIP), a member of the phosphatidylethanolamine binding protein (PEBP) family, is widely expressed in normal human tissues, highlighting its role in various physiologic processes [25], but is considered to be a metastasis suppressor in cancer, being its loss or reduced expression associated with malignancy and prognosis in many types of metastatic and aggressive cancers [26–29]. Recently, a study showed that snail, a mediator of the EMT, can inhibit RKIP transcription and negatively correlates with RKIP levels in tumors [30]. Additionally, another study reported that RKIP inhibition in cervical cancer is associated with higher tumor aggressive behavior and resistance to cisplatin therapy [31]. Our finding showed that downregulation of RKIP induces EMT and contributes to cisplatin resistance. We further establish miR-27a-RKIP as another important pathway regulating EMT and chemoresistance of lung adenocarcinoma cells.

Chemoresistance is a major issue of treatment in the majority of human tumors, including lung cancer. Thus, detecting rationale biomarkers to predict chemotherapy sensitivity and screening for targets to overcome resistance are significant for cancer therapy. A specific miRNA can affect simultaneously the expression of proteins involved in multiple cellular pathways, potentially serving as better therapeutic target or biomarker for clinical outcome than single proteins. In fact, several miRNAs, including miR-21, miR-10b and miR-125b, have been used as predictors of chemoresistance in cancers [32–34]. Herein, our observation that increased miR-27a expression is associated with chemotherapy resistance, and poor patient prognosis may provide surrogates to predict the chemotherapeutic sensitivity for lung adenocarcinoma.

## Conclusions

In conclusion, we have reported the altered expression of miR-27a in human lung adenocarcinoma cell lines with different sensitivities to cisplatin, and have shown that miR-27a could modulate cisplatin resistance and metastasis in these cells by targeting RKIP. Therefore, targeting miR-27a-RKIP interaction may be a potential strategy for reversing chemoresistance in human lung adenocarcinoma.

## Materials and methods

### Cell culture and transfection

Human lung adenocarcinoma A549 cells were purchased from the American Type Culture Collection and cultured in RPMI 1640 medium containing 10% fetal bovine serum (FBS) with 100 μg/ml penicillin /streptomycin at 37°C with 5% CO_2_. The cisplatin-resistant A549 cell line (A549/CDDP) was established and preserved in a 40 μmol/L final concentration of cisplatin in our laboratory.

miR-27a mimics and negative control mimics (NC), miR-27a inhibitors (anti-miR-27a) and negative control inhibitors (anti-NC) and RKIP siRNAs were synthesized by GenePharma Company (Shanghai, China). Transfection was performed with Lipofectamine 2000 (Invitrogen, CA, USA) according to the manufacturer’s protocol. Total RNA and protein were prepared 48 h after transfection and were used for qRT-PCR or Western blot analysis.

### Tissue samples

A total of 30 lung adenocarcinoma tissues were collected from patients with advanced lung adenocarcinoma who received chemotherapy at Yinzhou People’s Hospital (Ningbo, China) between January 2009 and March 2010. Informed consent was obtained from all subjects and this study was approved by the Clinical Research Ethics Committee of Yinzhou People’s Hospital. Patients met all of the following criteria: primary lung adenocarcinoma; histological diagnosis of lung adenocarcinoma with at least 1 measurable lesion; clinical stage IIIB-IV; first-line chemotherapy either with cisplatin 100 mg/m^2^ and docetaxel 75 mg/m^2^ or cisplatin 100 mg/m^2^ and gemcitabine 1000 mg/m^2^ administered every 3 weeks for a maximum of 5 cycles. Samples were divided into “sensitive” (complete response or partial response) and “insensitive” (stable disease or progressive disease) groups according to the patient’s responses assessed via medical image analysis and detection of serum tumor markers after 4 or 5 cycles of cisplatin-based chemotherapy.

### RNA extraction and qRT–PCR

Total RNA was extracted from the cultured cells and the lung adenocarcinoma tissue specimens using Trizol Reagent (Invitrogen, CA, USA) according to the manufacturer’s protocol. The expression level of mature miR-27a was measured by TaqMan miRNA assays (Applied Biosystems, CA, USA) according to the provided protocol, U6 snRNA levels were used for normalization. RKIP expression was measured by SYBR green qPCR assay (Takara, Dalian, China) and GAPDH was used as an endogenous control.

### Western blot analysis

Protein extracts were prepared by a modified RIPA buffer with 0.5% sodium dodecyl sulfate (SDS) in the presence of proteinase inhibitor cocktail (Complete mini, Roche, Indianapolis, IN, USA). Polyacrylamide gel electrophoresis, tank-based transfer to Immobilon Hybond-C membranes (Amersham Biosciences) and immunodetection were performed with standard techniques. Antibodies to RKIP (catalog no. sc-28837), E-cadherin (catalog no. sc-8426), vimentin (catalog no. sc-32322) and β-actin (catalog no. sc-1616) were purchased from Santa Cruz Biotechnology, Inc. (Santa Cruz, CA). Signals were visualized with SuperSignal® West Pico chemoluminescent substrate (Pierce, Rockford, III, USA) by exposure to films.

### Cell viability assay

Cells were seeded into 96-well plates (2 × 10^3^ cells/well) directly or 24 hours after transfection and allowed to attach overnight. Freshly prepared cisplatin was then added with different final concentrations. Forty-eight hours later, cell viability was assessed via 3-(4,5-dimethylthiazol-2-yl)-2,5-diphenyl-tetrazolium bromide (MTT) assay as described previously [35].

### Transwell invasion assay

2 × 10^5^ cells were added into the upper chamber of the insert precoated with Matrigel (ECM gel, Sigma-Aldrich, St. Louis, MO). Cells were plated in medium without serum, and medium containing 10% fetal bovine serum in the lower chamber served as chemoattractant. After several hours of incubation, the cells that did not invade through the pores were carefully wiped out with cotton wool, and the filters were fixed by treatment with 95% ethanol for 30 minutes and stained with 0.2% crystal violet solution for 30 minutes. Invasive cells adhering to the undersurface of the filter were counted (five fields/chamber; 0.24 mm^2^/field) using an inverted microscope, and each experiment was repeated three times.

### Plasmid construction and luciferase reporter assay

Wild-type 3′untranslated region (3′UTR) of RKIP containing predicted miR-27a target sites were amplified by PCR from A549 cell genomic DNA. Primers used: Forward: GAT CTG CAG GGG TTA GCT TGG GGA CCT GAA C; Reverse: GAT CAT ATG AGA GTG ACA TAC TGA TGC CTA C. Mutant 3′UTRs were generated by overlap-extension PCR method. Both wild-type and mutant 3′UTR fragments were subcloned into the pGL3-control vector (Promega, Madison, WI) immediately downstream of the stop codon of the luciferase gene. DNA fragment coding RKIP protein was amplified by PCR from A549 cell cDNA, and cloned into pCMV-Myc expression vector (Clonetech, Mountain View, CA). Primers used: Forward: GCT GAA TTC ATG CCG GTG GAC CTC AGC AAG T; Reverse: CTG CTC GAG CTA CTT CCC AGA CAG CTG CTC G. For luciferase assay, the reporter plasmid was cotransfected with a control Renilla luciferase vector into A549 cells in the presence of either miR-27a or NC. After 48 h, cells were harvested, and the luciferase activity was measured using the Dual-Luciferase Reporter Assay System (Promega, Madison, WI, USA).

### Animal studies

Five-week-old female BALB/c nude mice were purchased from the Animal Center of Zhejiang University (Hangzhou, China). For *in vivo* chemosensitivity and metastasis assays, A549 cells ( infected with either the miR-27a-overexpressing lentivirus or the mock lentivirus) and A549/CDDP cells (infected with either the miR-27a-knockdown lentivirus-mediated antagomir or the antagomir-NC) were subcutaneously inoculated into nude mice (six per group, 1 × 10^6^ cells for each mouse). Tumor growth was examined every other day, and tumor volumes were calculated using the equation V = A × B^2^/2 (mm^3^), where A is the largest diameter and B is the perpendicular diameter. When the average tumor size reached ≈ 50 mm^3^, cisplatin was administered via intraperitoneal injection at a dose of 5 mg/kg, 1 dose every other day, with 3 doses in total. After 2 weeks, all mice were sacrificed. Transplanted tumors were excised, and tumor tissues were used to perform hematoxylin & eosin (H&E) staining. All research involving animal complied with protocols approved by the Zhejiang medical experimental animal care commission.

### Statistical analysis

Statistical analyses were performed using SPSS 16.0 software (SPSS Inc.). All data from 3 independent experiments were expressed as mean ± SD. Differences were assessed by two-tailed Student’s *t* test. *P <* 0.05 was considered statistically significant.

## Electronic supplementary material

Additional file 1: Table S1: miRNAs differentially expressed in the A549/CDDP and A549 cell lines. (DOC 38 KB)

Additional file 2: Figure S1: miR-27a regulates EMT and cisplatin resistance in H1395 and H1299 cells. (A) The expression of miR-27a was examined by qRT-PCR. U6 small nuclear RNA was used as an internal control. (B) Western blotting was used to detect E-cadherin and vimentin expression, β-actin was used as an internal control. (C and D) MTT assay was used to measure cisplatin sensitivity. Data are means of three separated experiments ± SD; *P<0.05. (TIFF 1 MB)
