# Supplementary material for: miR-27a regulates cisplatin resistance and metastasis by targeting RKIP in human lung adenocarcinoma cells
Source: Mol Cancer. 2014 Aug 16;13:193. doi: 10.1186/1476-4598-13-193 (PMC4158130; doi:10.1186/1476-4598-13-193)
Supplement: Supplementary file 1 — Additional file 1: Table S1: miRNAs differentially expressed in the A549/CDDP and A549 cell lines. (DOC 38 KB) [file 12943_2014_1397_MOESM1_ESM.doc]

Table S1 miRNAs differentially expressed in the A549/CDDP and A549 cell lines

| miRNA | Up- or down-regulation in A549/CDDP | Mean fold change in A549/CDDP(log_2_) |
| --- | --- | --- |
| hsa-miR-134 | down | -3.94 |
| hsa-miR-200b | down | -3.49 |
| Has-miR-495 | down | 3.12 |
| hsa-miR-379 | down | -2.55 |
| hsa-miR-194 | down | -2.11 |
| hsa-miR-376a | down | -2.03 |
| hsa-miR-127 | down | -1.66 |
| hsa-miR-23a | Up | 1.53 |
| hsa-miR-125a | Up | 1.76 |
| hsa-miR-324 | Up | 1.80 |
| hsa-miR-100 | Up | 1.84 |
| hsa-miR-99a | Up | 1.92 |
| hsa-miR-27a | Up | 2.48 |
