# Supplementary figures and images for: miR-27a regulates cisplatin resistance and metastasis by targeting RKIP in human lung adenocarcinoma cells
Source: Mol Cancer. 2014 Aug 16;13:193. doi: 10.1186/1476-4598-13-193 (PMC4158130; doi:10.1186/1476-4598-13-193)

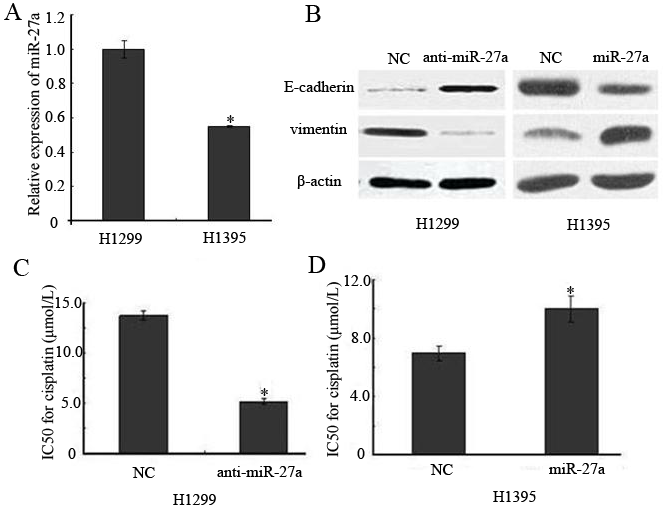

Supplement: Supplementary file 2 — Additional file 2: Figure S1: miR-27a regulates EMT and cisplatin resistance in H1395 and H1299 cells. (A) The expression of miR-27a was examined by qRT-PCR. U6 small nuclear RNA was used as an internal control. (B) Western blotting was used to detect E-cadherin and vimentin expression, β-actin was used as an internal control. (C and D) MTT assay was used to measure cisplatin sensitivity. Data are means of three separated experiments ± SD; *P<0.05. (TIFF 1 MB) [file 12943_2014_1397_MOESM2_ESM.tiff]
